# Estimating the public health impact of a national guideline on cervical cancer screening: an audit study of a program in Campinas, Brazil

**DOI:** 10.1186/s12889-019-7846-2

**Published:** 2019-11-08

**Authors:** Diama Bhadra Vale, Talita Lourenço Menin, Joana Froes Bragança, Julio Cesar Teixeira, Lucas Almeida Cavalcante, Luiz Carlos Zeferino

**Affiliations:** Department of Gynecology and Obstetrics, Faculty of Medical Sciences, University of Campinas, Rua Alexander Fleming 101, Campinas, CEP 13083-790 Brazil

## Abstract

**Background:**

A Brazilian guideline on cervical cancer screening was released in 2011. The objective was to verify changes in screening indicators around this period.

**Methods:**

An audit study which sample was all screening tests performed by the public health system of Campinas city from 2010 to 2016. Variables were absolute tests numbers, excess tests, intervals and results, by age. For trend analysis was used Cochran-Armitage × 2 and linear regression.

**Results:**

Were carried out 62,925 tests in 2010 and 43,523 tests in 2016, a tendency at a reduction (*P* = 0.001). Excess tests were higher than 50% over the years, with a tendency at a reduction (*P* < 0.001). Tests performed on women under 25 ranged from 20.2 to 15.4% in the period (*P* < 0.001), while in the 25–64 years age-group, it ranged from 75.1 to 80.2% (*P* < 0.001). In 2010 the most frequent interval was annual (47.5%) and in 2016 biennial (34.7%). There was a tendency at a reduction in the proportion of tests performed at the first time and those with an annual interval (P < 0.001), and also a tendency at an increase in tests with intervals equal to or greater than biannual (P < 0.001). We observed a tendency at a reduction in LSIL and HSIL-CIN2 results (*P* = 0.04 and *P* = 0.001, respectively), and a tendency at an increase in HSIL-CIN3 result (*P* = 0.02).

**Conclusion:**

The proportion of cervical cancer screening tests performed out of the recommendation showed a significant reduction in the period. This indicates a tendency to align cervical cancer screening in Campinas with the standards recommended.

## Background

Cervical cancer is the third most common cancer in Brazilian women, although a significant disparity in rates is observed due to different levels of social and economic development [1–3]. Prevention is possible through vaccination and screening of the target population, identifying women with precursor lesions and promoting timely treatment [4]. In Brazil, screening has been encouraged since the late ‘80s. However, there is no policy to ensure a routine invitation to the target population. In this scenario, screening is opportunistic, that is, tests are only performed when women access the health system for different reasons.

The consequence of the current screening model is that most tests are performed when a woman seeks gynaecological or obstetric care. In this way, tests are usually performed in the same women group (overscreening), while others do not participate in screening and are at higher risk of developing cervical cancer. This opportunistic screening strategy has limited efficiency [4–6].

In the last decades, the expansion of the health system in Brazil has enabled an increased provision of public health programs, particularly in primary health care (PHC), where screening is performed. From 2000 to 2010, the family health strategy program (PHC) increased coverage from 13.2 to 120.2 million people (7.8 to 58.5% of the population) [7]. In 2006 the first attempt for a national cervical cancer screening guideline was made in a technical report [8]. Only in 2011, a national policy for screening was established with a definite recommendation of services according to the level of care, how to obtain test quality control and how to manage abnormal results. For the target population from 25 to 64 years old, the recommendation for the screening test was cytology at a three-year interval after two annual negative results [9]. The guideline was updated in 2016, maintaining these recommendations [4]. The main limitation of the guideline is that it does not suggest an invitation strategy to reach the target population, preserving the opportunistic character of the program.

The guideline of 2011 was part of a national policy for the prevention, diagnosis, and treatment of cervical cancer. Cities were encouraged to adopt the guideline, and specific financial support was established to guarantee routine screening and early diagnosis [10].

In recent years, there has been a decrease in the absolute number of screening tests in the city of Campinas, in São Paulo state, Brazil. In 2010, about 64,000 tests were performed, while in 2016 only about 37,000 tests [11]. This reduction may be due to a change in the profile of the woman performing the test, in the behaviour of the professional performing the collection, or in the way the screening is being offered. In the same period, the epidemiologic profile did not change. Indeed, by that time, there was an intensification of the policies for cervical cancer control in Brazil, following the release of the national recommendation. In this way, is possible that this decrease would be related to modifications in how screening was delivered. The objective of this study is to evaluate if there was a tendency to align cervical cancer screening in Campinas with the standards recommended by the national guideline.

## Methods

The paper is an audit study of trend evaluation of screening indicators in Campinas, covering all screening tests performed by the public health system from 2010 to 2016. The data system only registers women screened. We do not have access to women out of the programme. Screening tests (cytology) were taken at primary health care, and all analysed at the university laboratory of the Women’s Hospital Prof. Dr. José Aristodemo Pinotti (CAISM/Unicamp).

Campinas is a city in São Paulo state with a high Human Development Index (0.805). The population of almost 1.2 million is mostly urban. The estimation is that about 50% of the population has access to private care, which means that at least 600,000 people exclusively rely on the public health system. HPV vaccine started in 2014 in Brazil to girls from 9 to 14 years old, so those girls were not yet in the target group for screening.

The study subjects were all the women who underwent the tests in this period. The instrument of data collection was the “Identification and Results Form”, which is divided into two parts. In the first part, there is the registration of the place of care, the identification of the patients, the relevant clinical information, time interval since the previous test, the purpose of the test (screening, control after abnormal result or control after treatment), and age. The second part is the data on sample quality and results. The first part is filled in by the examiner (doctor, nurse or nursing technician) and the second part by the cytologist in the laboratory. Completely filled-out sheets are optically read, and their information goes to the hospital information system, that provides aggregated data reports in Excel® tables.

Interval since the previous test was filled in by the examiner as nearly one, two, three or more than three years. In the clinical practice, it is recorded as rounded up to the nearest whole number. For example, a test done at 34 months is coded as three years interval.

Some tests were excluded: those whose purpose was identified as a previous abnormal test (control tests), post-treatment control, and those whose time-interval was less than one year (not qualified as screening tests). The variables analysed were: year, age, the time interval between tests (annual, biennial, triennial or greater than three years), and cytological results.

The classification of the results followed Bethesda [12] as: normal, atypical squamous cells of undetermined significate (ASC), low-grade squamous intraepithelial lesion (LSIL), high-grade squamous intraepithelial lesion (HSIL), atypical glandular cells (AGC), adenocarcinoma in situ (AIS), invasive squamous cell carcinoma (SCC), adenocarcinoma and other neoplasms. The aggregated data report does not differentiate “ASC possibly non-neoplastic” from “ASC when high-grade intraepithelial lesion cannot be excluded” (ASC-US or ASC-H). The laboratory distinguishes between high-grade squamous intraepithelial lesions suggestive of cervical intraepithelial neoplasia grade 2 or 3 (HSIL-CIN2/CIN3).

The study of van Ballegooijen et al. [13] was used as the reference for the calculation of excess tests. Excess tests were all those tests out of the national recommendation: those in women aged under 25 or older than 64 years old, and those repeated less than three years since the last test. The formula for the calculation consists of [2/3 (b-a) + 1 / 2c + d]. Being ‘a’ the first test performed by the woman; ‘b’, tests with an interval of 1 year; ‘c´ tests with an interval of 2 years; ‘d’ all tests performed outside the target age group (25 to 64 years). In the formula, the two first annually tests were excluded by “(b-a)”.

The variables were analysed by their frequencies. For trend analysis over the years, the Cochran-Armitage x^2^ (Z) and linear regression analysis (T) was used. When the statistic (Z or T) was positive (+), it indicated a tendency at an increase of the number of tests, and when negative (−) it indicated a tendency at a reduction of the number of tests. The significance level adopted was 5%, that is, *P* < 0.05. Analyses were performed through the SAS Program version 9.14 for Windows.

## Results

There was a significant tendency towards a reduction in the number of tests between 2010 and 2016 (62,925 and 43,523, respectively) (*P* = 0.001). Also, excess tests were higher than 50%, and over the years, a significant tendency towards a reduction was identified (*P* < 0.001) (Table 1).

**Table 1:** Total amount of cervical cancer screening tests and proportion of excess tests in Campinas

	2010	2011	2012	2013	2014	2015	2016	Tendency/ *P*-value
Total of tests	62,925	58,834	60,160	50,771	53,438	48,697	43,523	T = (−)6.54 P = 0.001
Excess tests	59%	58%	57%	56%	55%	53%	50%	T = (−)8.96 *P* < 0.001

*T* Test statistic, *P P*-value

Table 2 shows the proportion of the tests according to the screened age-group. Tests performed on women under 25 corresponded to 20.2% in 2010 and 15.4% in 2016, a significant tendency at a reduction (P < 0.001). In the 25–64 years age-group, an increase in the number of tests was observed (*P* < 0.001).

**Table 2:** Cervical cancer screening tests in Campinas according to the age of the women

	2010	2011	2012	2013	2014	2015	2016	Tendency/ *P*-value
< 25 years	12,697 20.18%	11,507 19.56%	10,981 18.25%	9060 17.84%	9541 17.85%	8214 16.87%	6708 15.41%	Z = (−)22.4 *P* < 0.001
25–64 years	47,275 75.13%	44,449 75.55%	46,036 76.52%	38,975 76.77%	41,235 77.16%	38,107 78.25%	34,882 80.15%	Z = 21.2 P < 0.001
> 64 years	2953 4.69%	2878 4.89%	3143 5.22%	2736 5.39%	2662 4.98%	2376 4.88%	1933 4.44%	Z = (−)1.3 *P* = 0.200
Total	62,925	58,834	60,160	50,771	53,438	48,697	43,523	

*Z* Test statistic, *P P*-value

Table 3 shows the proportion of tests according to the time interval. First-time examinations ranged from 6.1 to 7.0% in the period. In 2010 the most frequent interval was the annual (47.5%) and in 2016 the biennial (34.7%). There was a significant tendency at a reduction of the tests performed for the first time and those with an annual interval (*P* < 0.001), and a significant tendency at an increase of the tests with intervals equal to or higher than biannual (*P* < 0.001).

**Table 3:** Cervical cancer screening tests in Campinas according to the interval of the tests

Year/ Interval	2010	2011	2012	2013	2014	2015	2016	Tendency/ *P*-value
First test	4424 7.03%	4109 6.98%	3963 6.59%	3304 6.51%	3575 6.69%	3266 6.71%	2635 6.05%	Z = (−)5.75 P < 0.001
1 year	29,865 47.46%	26,113 44.38%	26,233 43.61%	19,977 39.35%	19,886 37.21%	17,096 35.11%	13,169 30.26%	Z = (−)67.26 P < 0.001
2 years	16,437 26.12%	16,257 27.63%	16,590 27.58%	15,754 31.03%	16,850 31.53%	15,687 32.21%	15,100 34.69%	Z = 36.79 P < 0.001
3 years	5353 8.51%	5420 9.21%	5837 9.70%	5220 10.28%	5806 10.86%	5714 11.73%	5934 13.63%	Z = 30.01 P < 0.001
> 3 years	5212 8.28%	5166 8.78%	5641 9.38%	5082 10.01%	5791 10.84%	5567 11.43%	5478 12.59%	Z = 28.15 P < 0.001
Not informed	1634 2.60%	1769 3.01%	1896 3.15%	1434 2.82%	1530 2.86%	1367 2.81%	1207 2.77%	Z = (−)0.05 *P* = 0.959
Total	62,925	58,834	60,160	50,771	53,438	48,697	43,523	

*Z* Test statistic, *P P*-value

Table 4 shows the distribution of tests according to the results. Normal results corresponded to almost all the tests, varying from 97.0 to 98.0% in the period. Among the abnormal results, the most frequent one was ASC (1.1 to 1.6%), followed by LSIL (0.3 to 0.6%). A significant tendency towards a reduction in the LSIL and HSIL-CIN2 results (*P* = 0.04 and *P* = 0.001, respectively), and a significant tendency towards an increase in the HSIL-CIN3 result was observed (*P* = 0.02).

**Table 4:** Cervical cancer screening tests in Campinas according to the results

	2010	2011	2012	2013	2014	2015	2016	Tendency/ *P*-value
Normal	61,006 96.95%	57,380 97.53%	58,857 97.83%	49,728 97.95%	52,213 97.71%	47,459 97.46%	42,257 97.09%	Z = 1.30 *P* = 0.072
ASC	1031 1.64%	843 1.43%	630 1.05%	544 1.07%	723 1.35%	796 1.63%	712 1.64%	Z = 1.62 *P* = 0.106
LSIL	382 0.61%	263 0.45%	187 0.31%	134 0.26%	187 0.35%	173 0.36%	268 0.62%	Z = (−)2.07 *P* = 0.039
HSIL-CIN2	113 0.18%	98 0.17%	69 0.11%	36 0.07%	68 0.13%	46 0.09%	63 0.14%	Z = (−)3.28 P = 0.001
HSIL-CIN3	85 0.14%	46 0.08%	51 0.08%	32 0.06%	74 0.14%	57 0.12%	70 0.16%	Z = 2.32 P = 0.02
AGC	28 0.04%	23 0.04%	11 0.02%	7 0.01%	9 0.02%	12 0.02%	21 0.05%	Z = (−)0.96 *P* = 0.337
AIS	1 0.00%	4 0.01%	2 0.00%	0 0.00%	0 0.00%	0 0.00%	1 0.00%	Z = (−)1.46 *P* = 0.143
SCC	7 0.01%	1 0.00%	6 0.01%	8 0.02%	10 0.02%	5 0.01%	5 0.01%	Z = 1.21 *P* = 0.228
Adenoca	8 0.01%	9 0.02%	2 0.00%	5 0.01%	6 0.01%	1 0.00%	3 0.01%	Z = (−)1.68 *P* = 0.094
Other neoplasias	0 0.00%	0 0.00%	0 0.00%	0 0.00%	2 0.00%	1 0.00%	0 0.00%	Z = 1.36 *P* = 0.173
No result	264 0.42%	167 0.28%	345 0.57%	277 0.55%	146 0.27%	147 0.30%	123 0.28%	Z = (−)4.37 P < 0.001
Total	62,925	58,834	60,160	50,771	53,438	48,697	43,523	

*Z* Test statistic, *P P*-value, *ASC* atypical squamous cells of undetermined significate, *LSIL* low grade squamous intraepithelial lesion, *HSIL-CIN2* high grade squamous intraepithelial lesions suggestive cervical intraepithelial neoplasia grade 2, *HSIL-CIN3* high grade squamous intraepithelial lesions suggestive cervical intraepithelial neoplasia grade 3, *AGC* atypical glandular cells, *AIS* adenocarcinoma in situ, *Adenoca* Adenocarcinoma, *SCC* invasive squamous cell carcinoma

## Discussion

In this audit study of cervical cancer screening indicators in the city of Campinas, we observed a reduction in the proportion of excess tests from 2010 to 2016, which indicates better performance of the screening program. A significant reduction in the identification of transient lesions and a significant increase in truly precursor lesions were also observed. These results may indicate a positive effect of the implementation of the cervical cancer control policy [4].

Cervical cancer screening in Campinas is opportunistic. It is a common practice among these women to perform tests at short intervals and outside the recommended target group (over-screening). Besides, women without access to the health system do not participate in screening, leading to low coverage of the target population [6].

In this study, we observed that about 2/3 of the tests were performed at intervals lower than the recommended (three years). A reduction in the proportion of tests performed at a one-year interval was observed in the period, and also a tendency to spacing this interval to two or three years. An increase in the proportion of tests performed at intervals of more than three years may indicate a higher uptake of women who were outside the screening program. These results indicate compliance with the recommendations. Tests performed for the first time reduced, but also tests performed on women younger than 25 years reduced, indicating compliance with the recommended target age-group.

We observed a tendency at an increase of the proportion of tests in the target age-group of 25 to 64 years, with a tendency at a reduction of the proportion of tests in women under 25 years, although in this age-group this proportion remained high (15,4% of all tests in 2016). Screening women under 25 years have not shown an impact on cervical cancer mortality [14]. Also, the incidence of cervical cancer in young women is low [15], and screening efficiency is limited [16]. The observed decreasing tendency in the proportion of tests in young women may reveal more considerable attention to the target age group, where the impact of screening is better observed.

One consequence of performing screening tests in young women is the high proportion of LSIL results. LSIL represents the cytological manifestation of Papillomavirus (HPV) intracytoplasmic multiplication. Most of these lesions regress spontaneously, especially in young women [17]. Once identified by screening, they lead to repeated examinations and overuse procedures, such as biopsies, colposcopies, and excisional techniques, with recognised obstetric morbidity [18, 19]. Even HSIL-CIN2 in young women seems to behave similarly to LSIL, with high rates of spontaneous regression [20, 21]. The reduction in the proportion of LSIL and HSIL-CIN2 results observed in our study, probably due to a decrease in the proportion of screened young women, indicates an improvement in program efficiency.

HSIL-CIN3 is considered the true precursor lesion in all age groups [17]. During the implementation of an organised screening program, an increase in the identification of precursor lesions is expected, which are the prevalent lesions that have not yet been identified. With time, as these lesions are treated, the number of precursor lesions identified falls, as well as the number of invasive cancers [4].

In our study, we observed a tendency at an increase of the proportion of HSIL-CIN3, although at a very low frequency (about 0.1% of the total number of exams), and without a significant change in the outcome of invasive results. Possibly, this result is associated to the increase of the number women of the target population, either by increasing the coverage of women between 25 and 64 years-old or by the rise in the number of tests performed in women with more than three-year interval. This would indicate an improvement in program efficiency. If this hypothesis is correct, in a few years a reduction of precursor lesions may be observed, with a consequent decrease in the incidence of cancer.

The main result of this study was a significant reduction in the tests performed in excess. There is a suggestion that opportunistic screening is not as cost-effective as organised screening. In opportunistic screening, the reduction in incidence and mortality rates is not as pronounced as in organised screening, and health resources are poorly optimised [5, 6, 18, 22]. Reducing the number of excess tests means reducing screening program costs and enhancing women’s access.

The implementation of an organised screening program is a complex task in public health. The critical component is an invitation system to the target population. This invitation would support equity on access and higher coverage. In low and middle-income regions, usually a population-based registry is absent, and screening is opportunistic. Other strategies towards an increase in the efficiency of the program should be developed.

In 2011 the Ministry of Health, in collaboration with the National Cancer Institute and the academy, all joined to improve screening performance by primary health care, which is provided by municipalities. The positive results showed in this study needs to be viewed in this context, as Campinas can be considered an index city in the implementation of public health programs. As a final result, reducing the number of unnecessary tests allows the entry of other individuals into the program, using the same resources. Additional efforts must be made to increase coverage of the target population.

The main strength of this study is the fact that it enacted a population-based assessment since all the tests performed by the public health in Campinas in the period could be analysed. In addition, it has benefited from the existence of a good quality information system. The study did not propose to analyse the data of private health care since it intended to evaluate only the performance of the public policies in the region. It is not possible to assess whether private care screening is also complying with the guidelines. We also do not know what is happening in women without access to the program, as it is an opportunistic screening, so our conclusion refers only to women included. Because data were aggregated and not linked to other information systems, it was not possible to confirm histopathological results. Since the period analysed was short (2010 to 2016), it is not yet possible to say whether the results will translate into improvements in incidence and mortality rates.

## Conclusions

Audits in health programs by using performance indicators are an essential step in quality control. In this study, we observed that the proportion of cervical cancer screening excess tests performed in the public health system of Campinas showed a significant reduction between 2010 and 2016. This result indicates a tendency to the adequacy of the program to the current guideline, towards an organised screening program.

## Data Availability

The data that support the findings of this study are available from the University Laboratory of the Women’s Hospital Prof. Dr. José Aristodemo Pinotti (CAISM/Unicamp), but restrictions apply to the availability of these data, which were used under license for the current study, and so are not publicly available. Data are however available from the authors upon reasonable request and with permission of both the laboratory and the county health secretary of Campinas.

## Abbreviations

Adenoca: Adenocarcinoma
AGC: Atypical glandular cells
AIS: Adenocarcinoma in situ
ASC: Atypical squamous cells of undetermined significate
ASC-H: ASC when high-grade intraepithelial lesion cannot be excluded
ASC-US: ASC possibly non-neoplastic
HPV: Human Papillomavirus
HSIL-CIN2: High-grade squamous intraepithelial lesions suggestive cervical intraepithelial neoplasia grade 2
HSIL-CIN3: High-grade squamous intraepithelial lesions suggestive cervical intraepithelial neoplasia grade 3
LSIL: Low-grade squamous intraepithelial lesion
P: *P*-value
SCC: Invasive squamous cell carcinoma
T: Test statistic
Z: Test statistic
